# Supplementary material for: miR766-3p and miR124-3p Dictate Drug Resistance and Clinical Outcome in HNSCC
Source: Cancers (Basel). 2022 Oct 27;14(21):5273. doi: 10.3390/cancers14215273 (PMC9655574; doi:10.3390/cancers14215273)
Supplement: Supplementary file 1 [file cancers-14-05273-s001.zip › Supplementary Figures.pptx]

## Slide 1
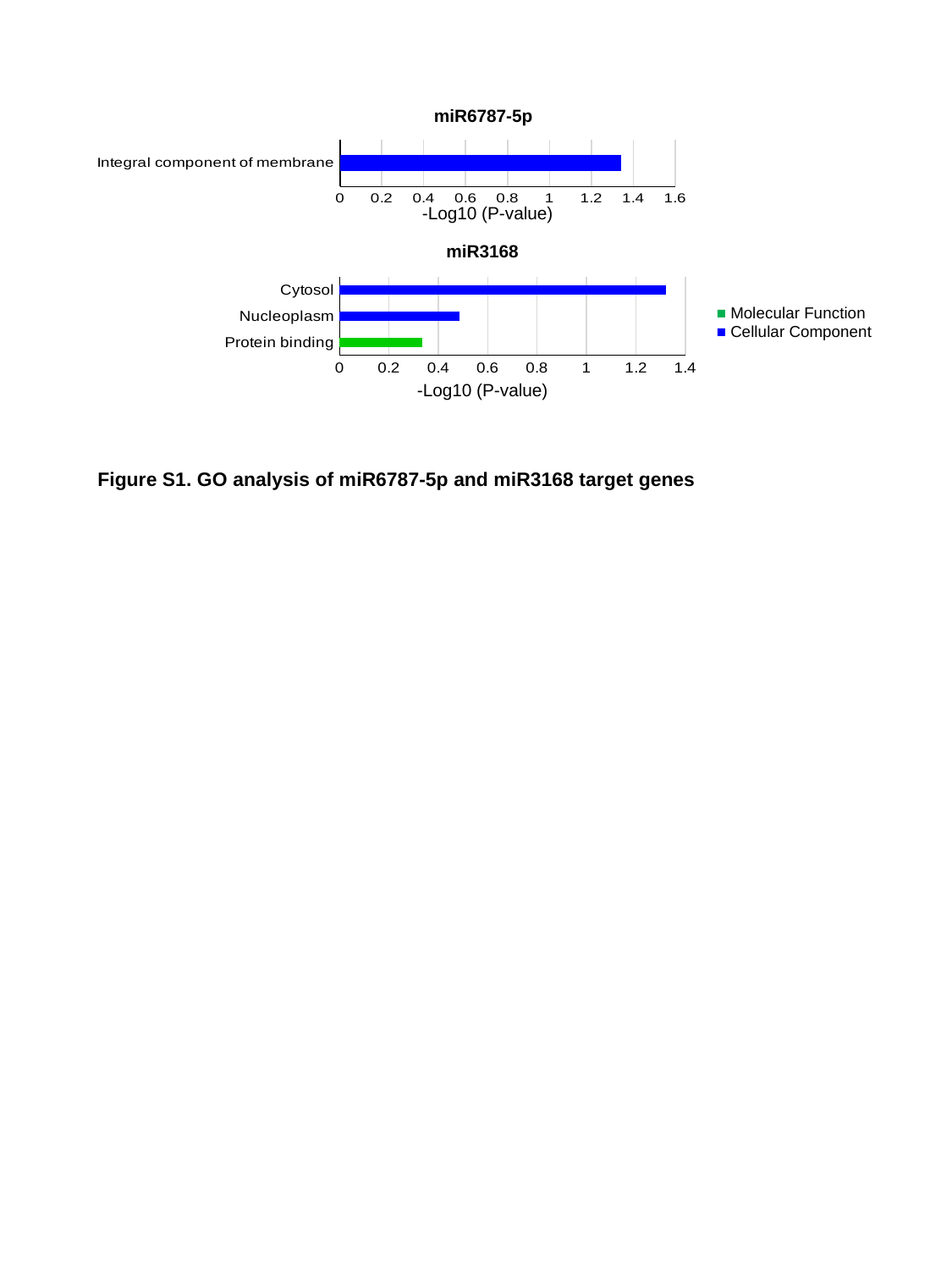

miR6787-5p
### Chart
| Category | |
|---|---|
| Integral component of membrane | 1.342109913552316 |-Log10 (P-value)
miR3168
### Chart
| Category | |
|---|---|
| Protein binding | 0.33656969552270705 |
| Nucleoplasm | 0.48679823534920486 |
| Cytosol | 1.3224542729610969 |■ Molecular Function
■ Cellular Component
-Log10 (P-value)
Figure S1. GO analysis of miR6787-5p and miR3168 target genes

## Slide 2
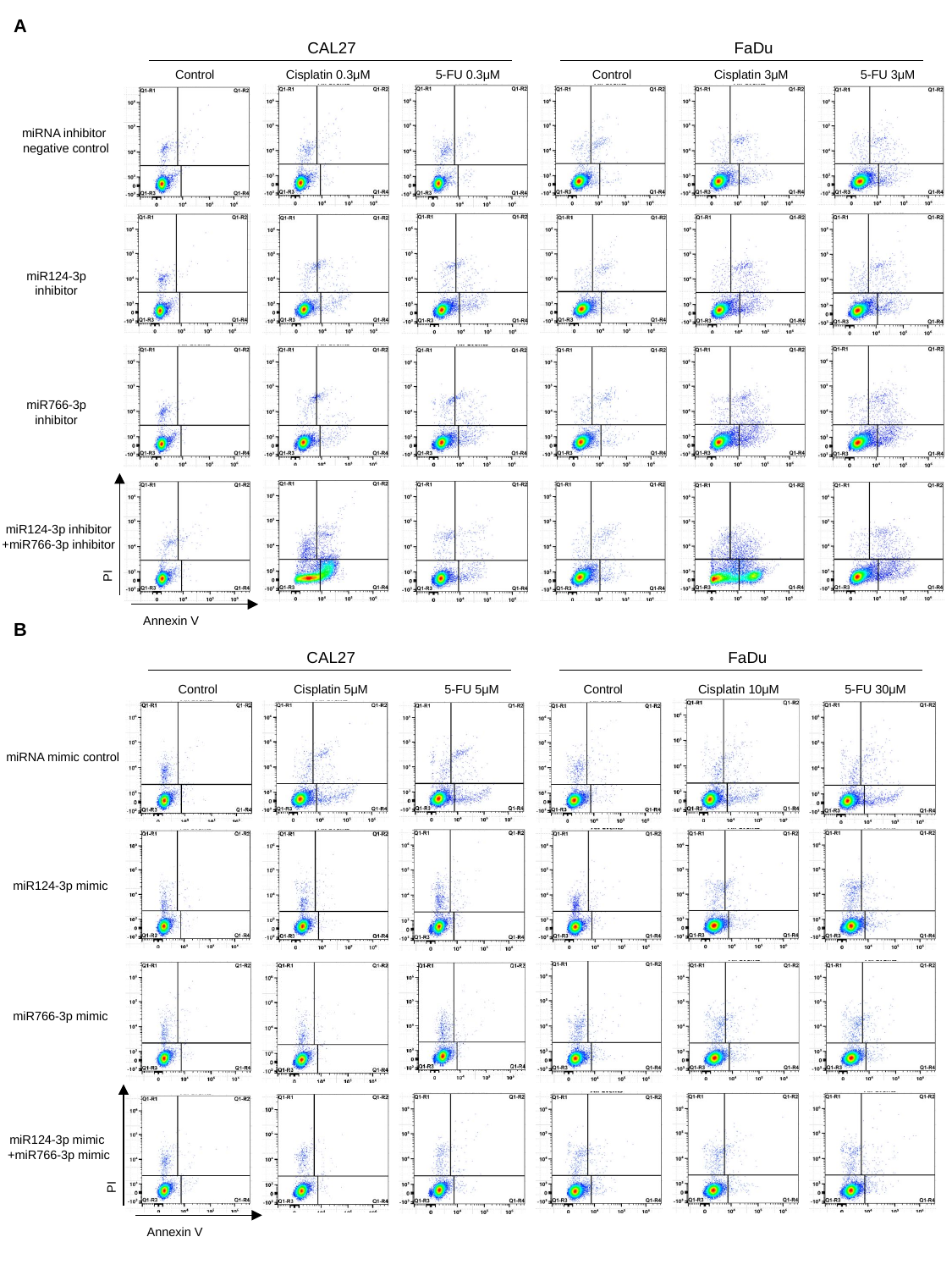

A
FaDu
CAL27
Control
Cisplatin 0.3μM
5-FU 0.3μM
Control
Cisplatin 3μM
5-FU 3μM
miRNA inhibitor
negative control
miR124-3p inhibitor
miR766-3p inhibitor
miR124-3p inhibitor
+miR766-3p inhibitor
PI
Annexin V
B
FaDu
CAL27
Control
Cisplatin 5μM
5-FU 5μM
Control
Cisplatin 10μM
5-FU 30μM
miRNA mimic control
miR124-3p mimic
miR766-3p mimic
miR124-3p mimic
+miR766-3p mimic
PI
Annexin V

## Slide 3
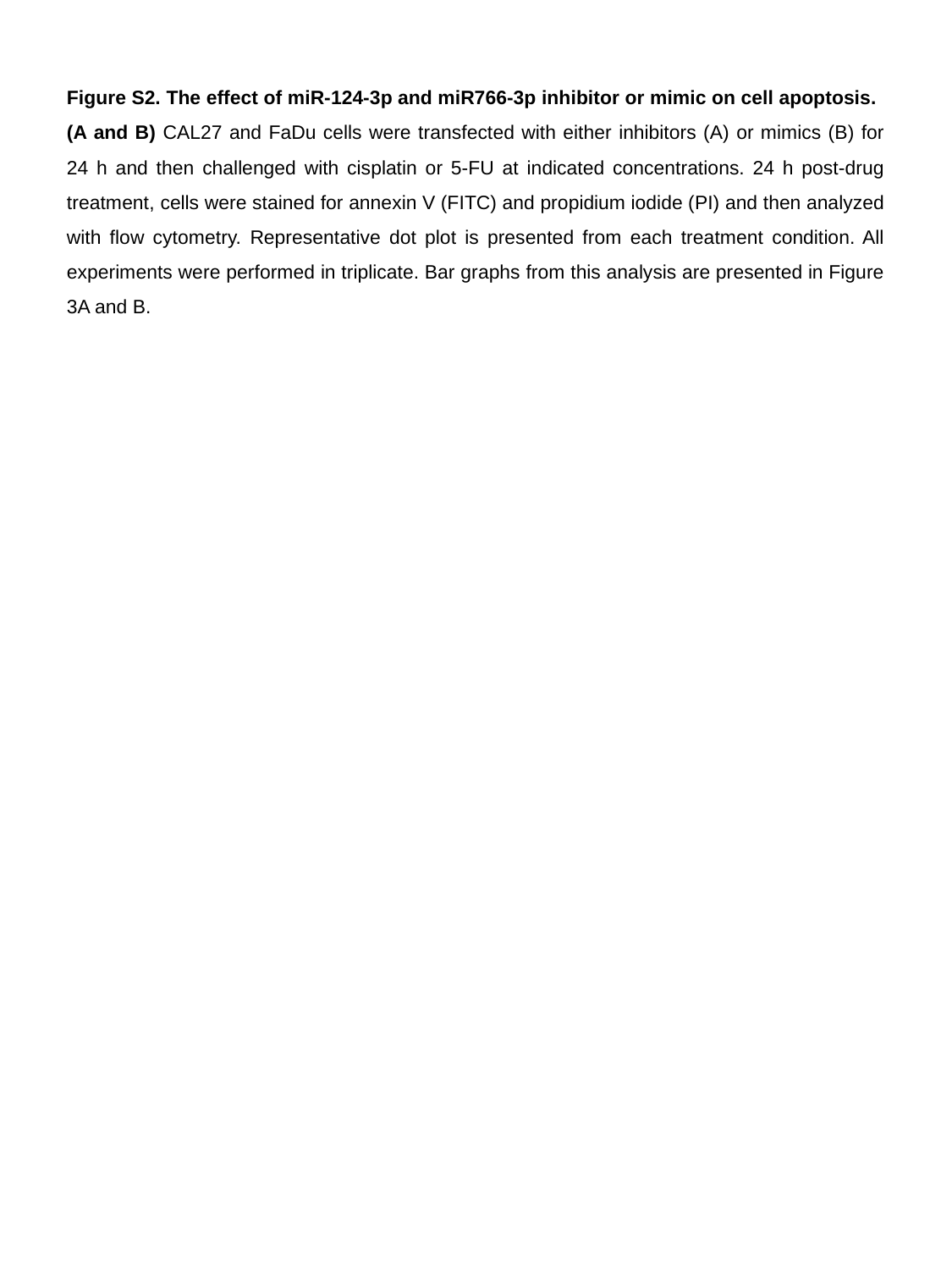

Figure S2. The effect of miR-124-3p and miR766-3p inhibitor or mimic on cell apoptosis.
(A and B) CAL27 and FaDu cells were transfected with either inhibitors (A) or mimics (B) for 24 h and then challenged with cisplatin or 5-FU at indicated concentrations. 24 h post-drug treatment, cells were stained for annexin V (FITC) and propidium iodide (PI) and then analyzed with flow cytometry. Representative dot plot is presented from each treatment condition. All experiments were performed in triplicate. Bar graphs from this analysis are presented in Figure 3A and B.

## Slide 4
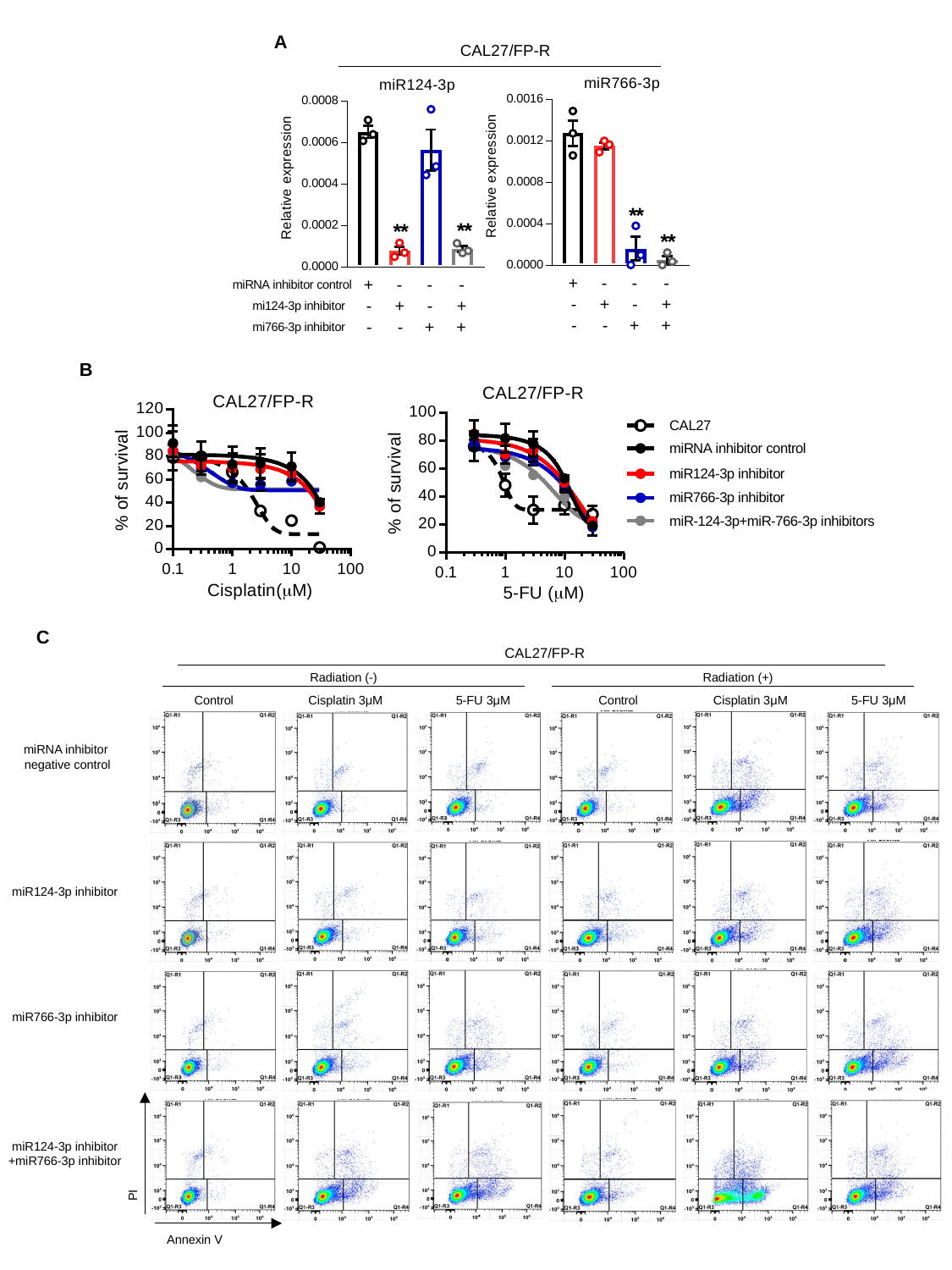

A
CAL27/FP-R
B
C
CAL27/FP-R
Radiation (-)
Radiation (+)
Control
Cisplatin 3μM
5-FU 3μM
Control
Cisplatin 3μM
5-FU 3μM
miRNA inhibitor
negative control
miR124-3p inhibitor
miR766-3p inhibitor
miR124-3p inhibitor
+miR766-3p inhibitor
PI
Annexin V

## Slide 5
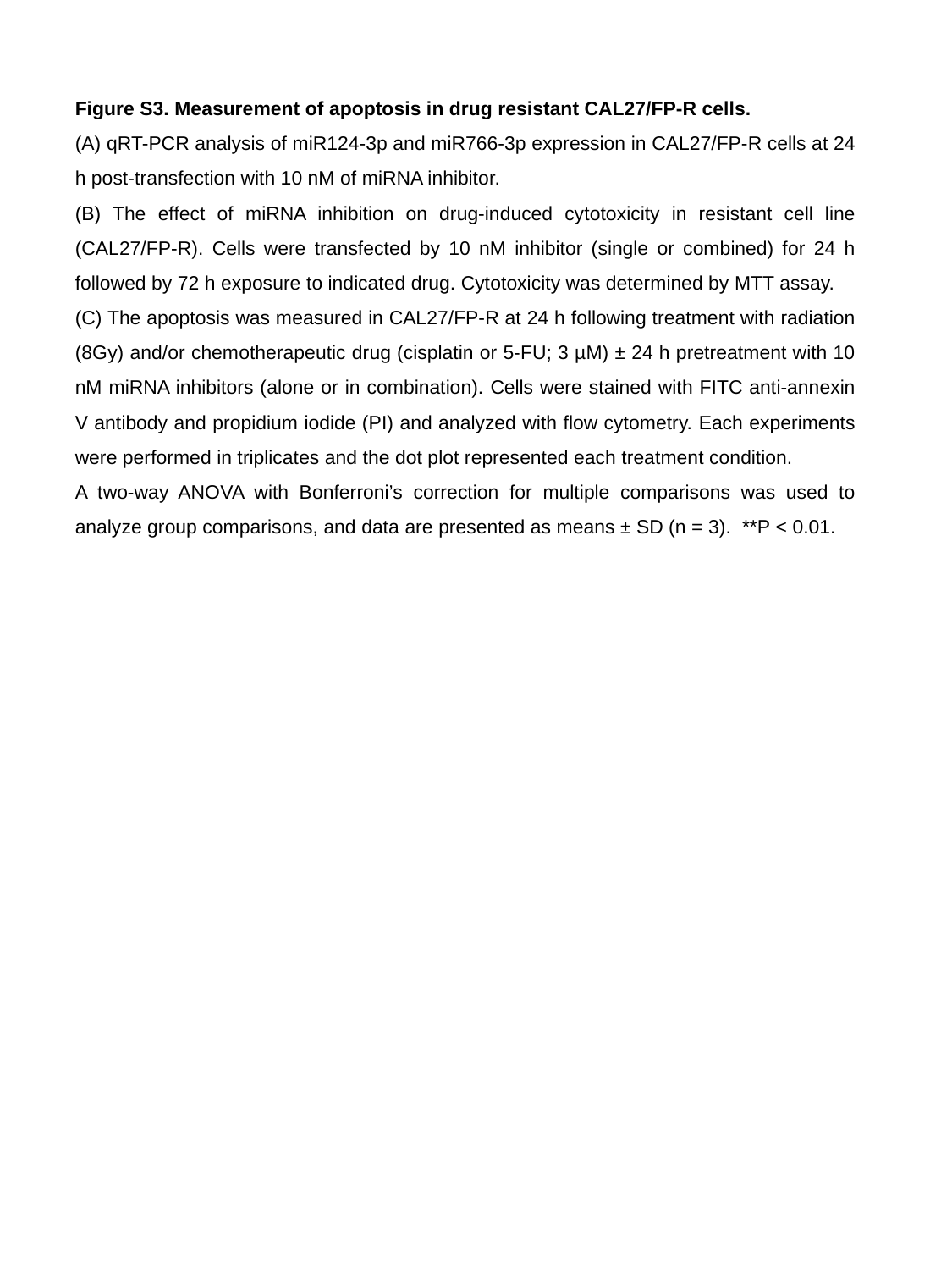

Figure S3. Measurement of apoptosis in drug resistant CAL27/FP-R cells.
(A) qRT-PCR analysis of miR124-3p and miR766-3p expression in CAL27/FP-R cells at 24 h post-transfection with 10 nM of miRNA inhibitor.
(B) The effect of miRNA inhibition on drug-induced cytotoxicity in resistant cell line (CAL27/FP-R). Cells were transfected by 10 nM inhibitor (single or combined) for 24 h followed by 72 h exposure to indicated drug. Cytotoxicity was determined by MTT assay.
(C) The apoptosis was measured in CAL27/FP-R at 24 h following treatment with radiation (8Gy) and/or chemotherapeutic drug (cisplatin or 5-FU; 3 µM) ± 24 h pretreatment with 10 nM miRNA inhibitors (alone or in combination). Cells were stained with FITC anti-annexin V antibody and propidium iodide (PI) and analyzed with flow cytometry. Each experiments were performed in triplicates and the dot plot represented each treatment condition.
A two-way ANOVA with Bonferroni’s correction for multiple comparisons was used to analyze group comparisons, and data are presented as means ± SD (n = 3). **P < 0.01.

## Slide 6
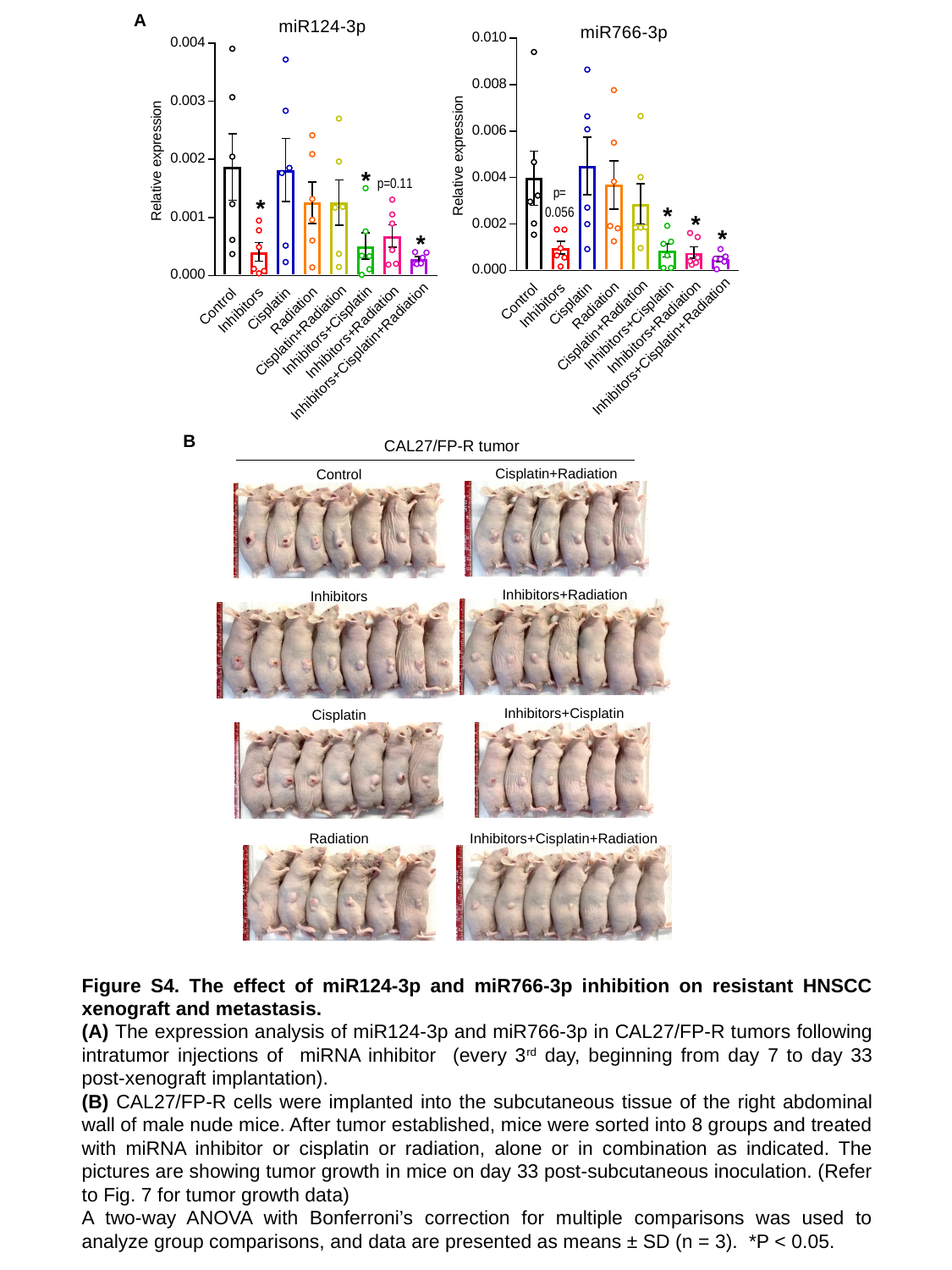

A
B
CAL27/FP-R tumor
Cisplatin+Radiation
Control
Inhibitors+Radiation
Inhibitors
Inhibitors+Cisplatin
Cisplatin
Inhibitors+Cisplatin+Radiation
Radiation
Figure S4. The effect of miR124-3p and miR766-3p inhibition on resistant HNSCC xenograft and metastasis.
(A) The expression analysis of miR124-3p and miR766-3p in CAL27/FP-R tumors following intratumor injections of miRNA inhibitor (every 3rd day, beginning from day 7 to day 33 post-xenograft implantation).
(B) CAL27/FP-R cells were implanted into the subcutaneous tissue of the right abdominal wall of male nude mice. After tumor established, mice were sorted into 8 groups and treated with miRNA inhibitor or cisplatin or radiation, alone or in combination as indicated. The pictures are showing tumor growth in mice on day 33 post-subcutaneous inoculation. (Refer to Fig. 7 for tumor growth data)
A two-way ANOVA with Bonferroni’s correction for multiple comparisons was used to analyze group comparisons, and data are presented as means ± SD (n = 3). *P < 0.05.

## Slide 7
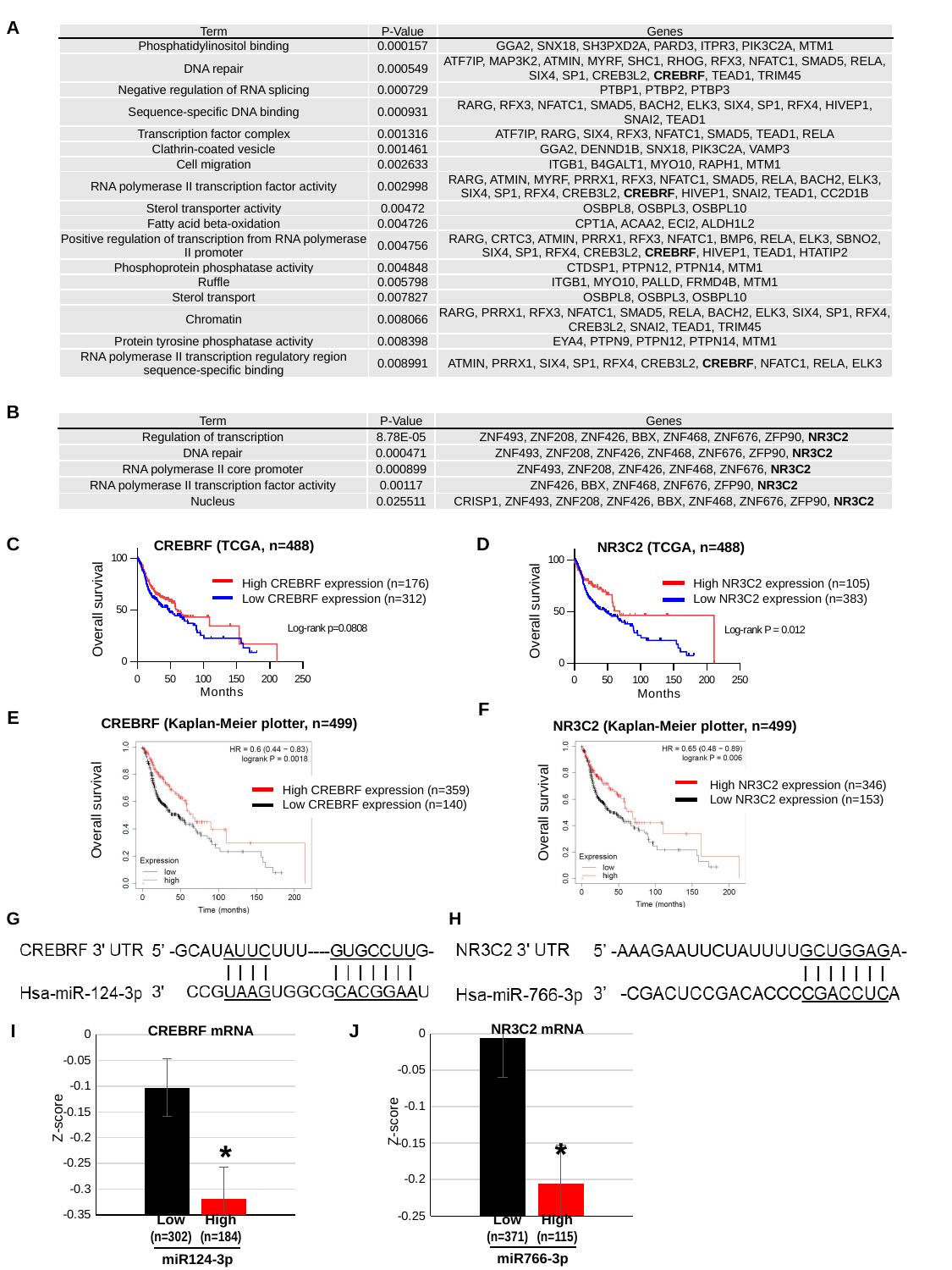

A
| Term | P-Value | Genes |
| --- | --- | --- |
| Phosphatidylinositol binding | 0.000157 | GGA2, SNX18, SH3PXD2A, PARD3, ITPR3, PIK3C2A, MTM1 |
| DNA repair | 0.000549 | ATF7IP, MAP3K2, ATMIN, MYRF, SHC1, RHOG, RFX3, NFATC1, SMAD5, RELA, SIX4, SP1, CREB3L2, CREBRF, TEAD1, TRIM45 |
| Negative regulation of RNA splicing | 0.000729 | PTBP1, PTBP2, PTBP3 |
| Sequence-specific DNA binding | 0.000931 | RARG, RFX3, NFATC1, SMAD5, BACH2, ELK3, SIX4, SP1, RFX4, HIVEP1, SNAI2, TEAD1 |
| Transcription factor complex | 0.001316 | ATF7IP, RARG, SIX4, RFX3, NFATC1, SMAD5, TEAD1, RELA |
| Clathrin-coated vesicle | 0.001461 | GGA2, DENND1B, SNX18, PIK3C2A, VAMP3 |
| Cell migration | 0.002633 | ITGB1, B4GALT1, MYO10, RAPH1, MTM1 |
| RNA polymerase II transcription factor activity | 0.002998 | RARG, ATMIN, MYRF, PRRX1, RFX3, NFATC1, SMAD5, RELA, BACH2, ELK3, SIX4, SP1, RFX4, CREB3L2, CREBRF, HIVEP1, SNAI2, TEAD1, CC2D1B |
| Sterol transporter activity | 0.00472 | OSBPL8, OSBPL3, OSBPL10 |
| Fatty acid beta-oxidation | 0.004726 | CPT1A, ACAA2, ECI2, ALDH1L2 |
| Positive regulation of transcription from RNA polymerase II promoter | 0.004756 | RARG, CRTC3, ATMIN, PRRX1, RFX3, NFATC1, BMP6, RELA, ELK3, SBNO2, SIX4, SP1, RFX4, CREB3L2, CREBRF, HIVEP1, TEAD1, HTATIP2 |
| Phosphoprotein phosphatase activity | 0.004848 | CTDSP1, PTPN12, PTPN14, MTM1 |
| Ruffle | 0.005798 | ITGB1, MYO10, PALLD, FRMD4B, MTM1 |
| Sterol transport | 0.007827 | OSBPL8, OSBPL3, OSBPL10 |
| Chromatin | 0.008066 | RARG, PRRX1, RFX3, NFATC1, SMAD5, RELA, BACH2, ELK3, SIX4, SP1, RFX4, CREB3L2, SNAI2, TEAD1, TRIM45 |
| Protein tyrosine phosphatase activity | 0.008398 | EYA4, PTPN9, PTPN12, PTPN14, MTM1 |
| RNA polymerase II transcription regulatory region sequence-specific binding | 0.008991 | ATMIN, PRRX1, SIX4, SP1, RFX4, CREB3L2, CREBRF, NFATC1, RELA, ELK3 |
B
| Term | P-Value | Genes |
| --- | --- | --- |
| Regulation of transcription | 8.78E-05 | ZNF493, ZNF208, ZNF426, BBX, ZNF468, ZNF676, ZFP90, NR3C2 |
| DNA repair | 0.000471 | ZNF493, ZNF208, ZNF426, ZNF468, ZNF676, ZFP90, NR3C2 |
| RNA polymerase II core promoter | 0.000899 | ZNF493, ZNF208, ZNF426, ZNF468, ZNF676, NR3C2 |
| RNA polymerase II transcription factor activity | 0.00117 | ZNF426, BBX, ZNF468, ZNF676, ZFP90, NR3C2 |
| Nucleus | 0.025511 | CRISP1, ZNF493, ZNF208, ZNF426, BBX, ZNF468, ZNF676, ZFP90, NR3C2 |
C
D
CREBRF (TCGA, n=488)
NR3C2 (TCGA, n=488)
High CREBRF expression (n=176)
Low CREBRF expression (n=312)
High NR3C2 expression (n=105)
Low NR3C2 expression (n=383)
F
E
CREBRF (Kaplan-Meier plotter, n=499)
NR3C2 (Kaplan-Meier plotter, n=499)
High NR3C2 expression (n=346)
Low NR3C2 expression (n=153)
High CREBRF expression (n=359)
Low CREBRF expression (n=140)
Overall survival
Overall survival
G
H
I
J
NR3C2 mRNA
CREBRF mRNA
### Chart
| Category | | |
|---|---|---|
### Chart
| Category | miR124-3p low | miR124-3p high |
|---|---|---|Z-score
Z-score
*
*
Low
(n=302)
High
(n=184)
Low
(n=371)
High
(n=115)
miR766-3p
miR124-3p

## Slide 8
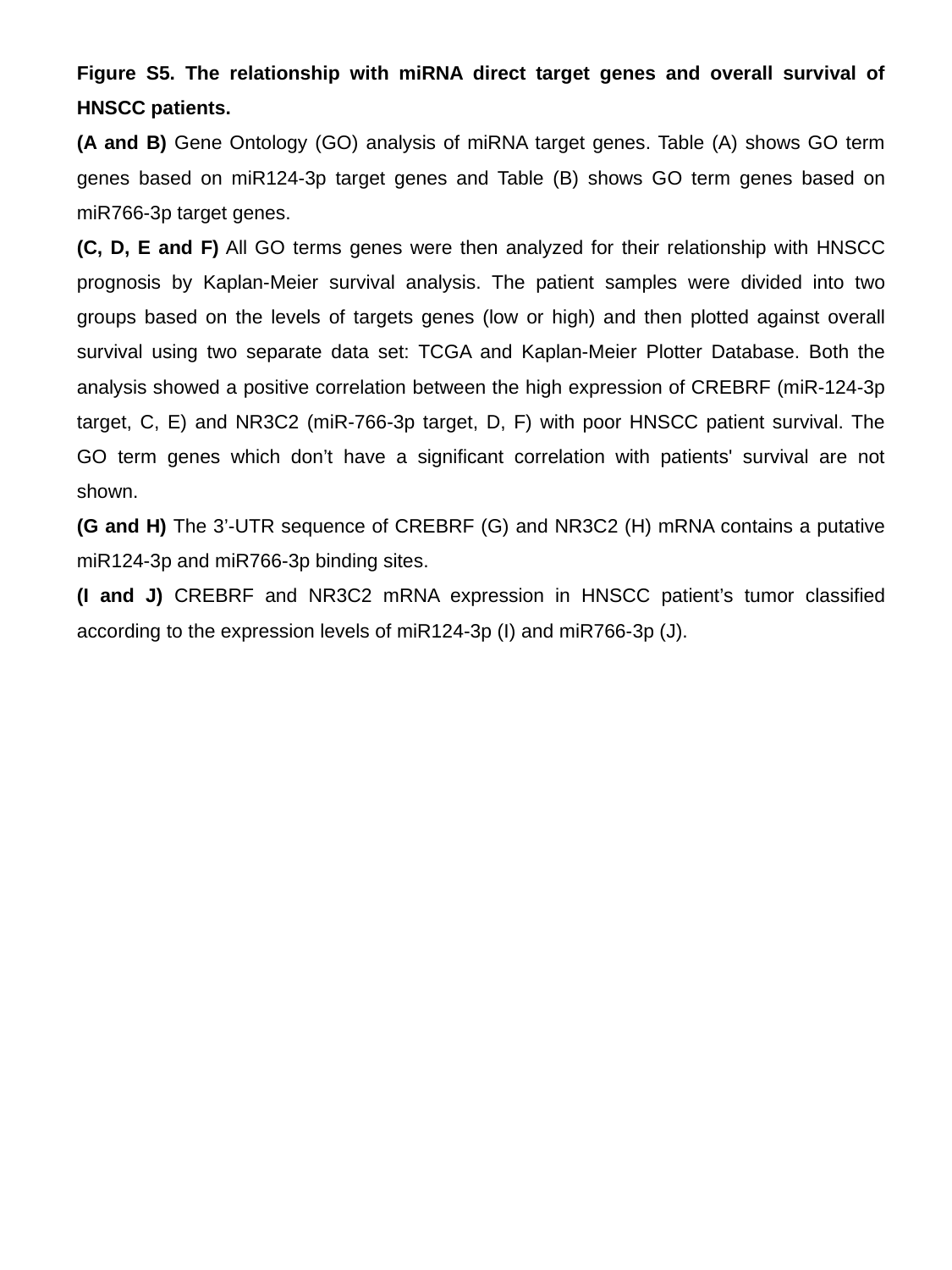

Figure S5. The relationship with miRNA direct target genes and overall survival of HNSCC patients.
(A and B) Gene Ontology (GO) analysis of miRNA target genes. Table (A) shows GO term genes based on miR124-3p target genes and Table (B) shows GO term genes based on miR766-3p target genes.
(C, D, E and F) All GO terms genes were then analyzed for their relationship with HNSCC prognosis by Kaplan-Meier survival analysis. The patient samples were divided into two groups based on the levels of targets genes (low or high) and then plotted against overall survival using two separate data set: TCGA and Kaplan-Meier Plotter Database. Both the analysis showed a positive correlation between the high expression of CREBRF (miR-124-3p target, C, E) and NR3C2 (miR-766-3p target, D, F) with poor HNSCC patient survival. The GO term genes which don’t have a significant correlation with patients' survival are not shown.
(G and H) The 3’-UTR sequence of CREBRF (G) and NR3C2 (H) mRNA contains a putative miR124-3p and miR766-3p binding sites.
(I and J) CREBRF and NR3C2 mRNA expression in HNSCC patient’s tumor classified according to the expression levels of miR124-3p (I) and miR766-3p (J).

## Slide 9
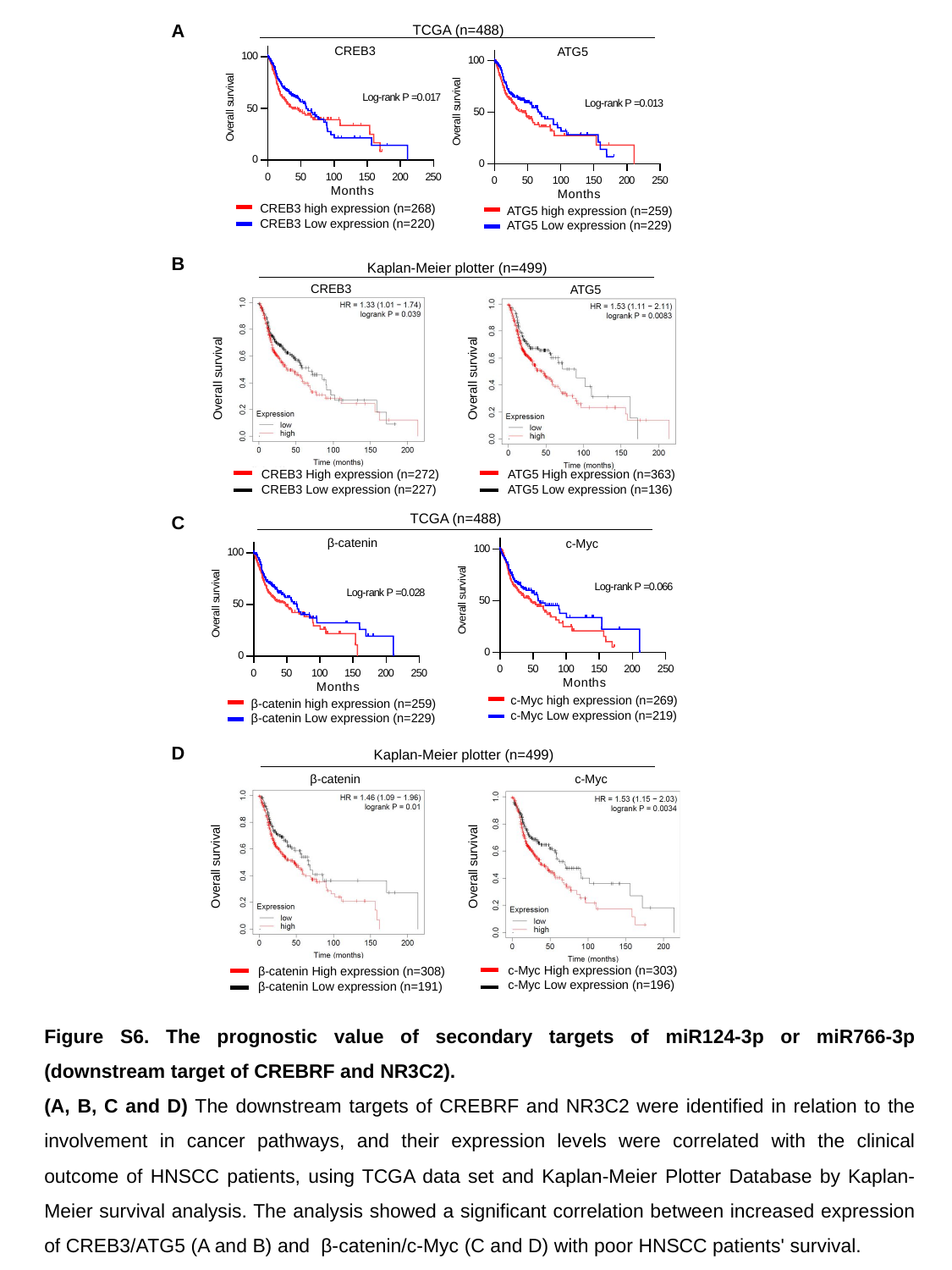

A
TCGA (n=488)
CREB3
ATG5
CREB3 high expression (n=268)
CREB3 Low expression (n=220)
ATG5 high expression (n=259)
ATG5 Low expression (n=229)
B
Kaplan-Meier plotter (n=499)
CREB3
ATG5
Overall survival
Overall survival
ATG5 High expression (n=363)
ATG5 Low expression (n=136)
CREB3 High expression (n=272)
CREB3 Low expression (n=227)
TCGA (n=488)
C
β-catenin
c-Myc
c-Myc high expression (n=269)
c-Myc Low expression (n=219)
β-catenin high expression (n=259)
β-catenin Low expression (n=229)
D
Kaplan-Meier plotter (n=499)
β-catenin
c-Myc
Overall survival
Overall survival
c-Myc High expression (n=303)
c-Myc Low expression (n=196)
β-catenin High expression (n=308)
β-catenin Low expression (n=191)
Figure S6. The prognostic value of secondary targets of miR124-3p or miR766-3p (downstream target of CREBRF and NR3C2).
(A, B, C and D) The downstream targets of CREBRF and NR3C2 were identified in relation to the involvement in cancer pathways, and their expression levels were correlated with the clinical outcome of HNSCC patients, using TCGA data set and Kaplan-Meier Plotter Database by Kaplan-Meier survival analysis. The analysis showed a significant correlation between increased expression of CREB3/ATG5 (A and B) and β-catenin/c-Myc (C and D) with poor HNSCC patients' survival.

## Slide 10
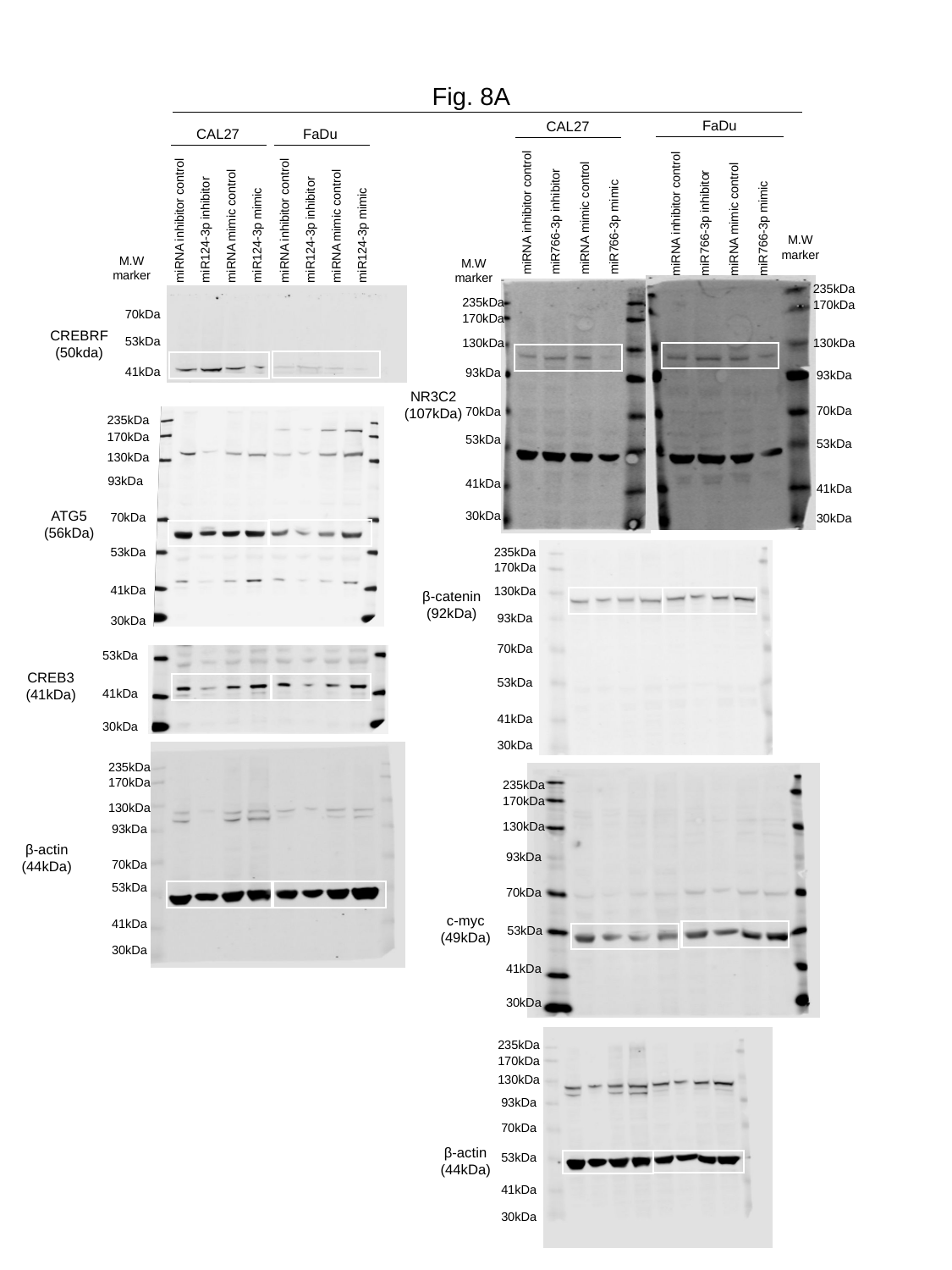

Fig. 8A
FaDu
CAL27
CAL27
FaDu
miRNA inhibitor control
miRNA inhibitor control
miRNA mimic control
miRNA mimic control
miRNA inhibitor control
miRNA inhibitor control
miR766-3p mimic
miR766-3p mimic
miR766-3p inhibitor
miR766-3p inhibitor
miRNA mimic control
miRNA mimic control
miR124-3p inhibitor
miR124-3p inhibitor
miR124-3p mimic
miR124-3p mimic
M.W marker
M.W marker
M.W marker
235kDa
235kDa
170kDa
70kDa
170kDa
CREBRF
(50kda)
53kDa
130kDa
130kDa
41kDa
93kDa
93kDa
NR3C2
(107kDa)
70kDa
70kDa
235kDa
170kDa
53kDa
53kDa
130kDa
93kDa
41kDa
41kDa
ATG5
(56kDa)
30kDa
70kDa
30kDa
235kDa
170kDa
130kDa
93kDa
70kDa
53kDa
41kDa
30kDa
53kDa
41kDa
β-catenin
(92kDa)
30kDa
53kDa
CREB3
(41kDa)
41kDa
30kDa
235kDa
235kDa
170kDa
130kDa
93kDa
70kDa
53kDa
41kDa
30kDa
170kDa
130kDa
93kDa
β-actin
(44kDa)
70kDa
53kDa
c-myc
(49kDa)
41kDa
30kDa
235kDa
170kDa
130kDa
93kDa
70kDa
β-actin
(44kDa)
53kDa
41kDa
30kDa

## Slide 11
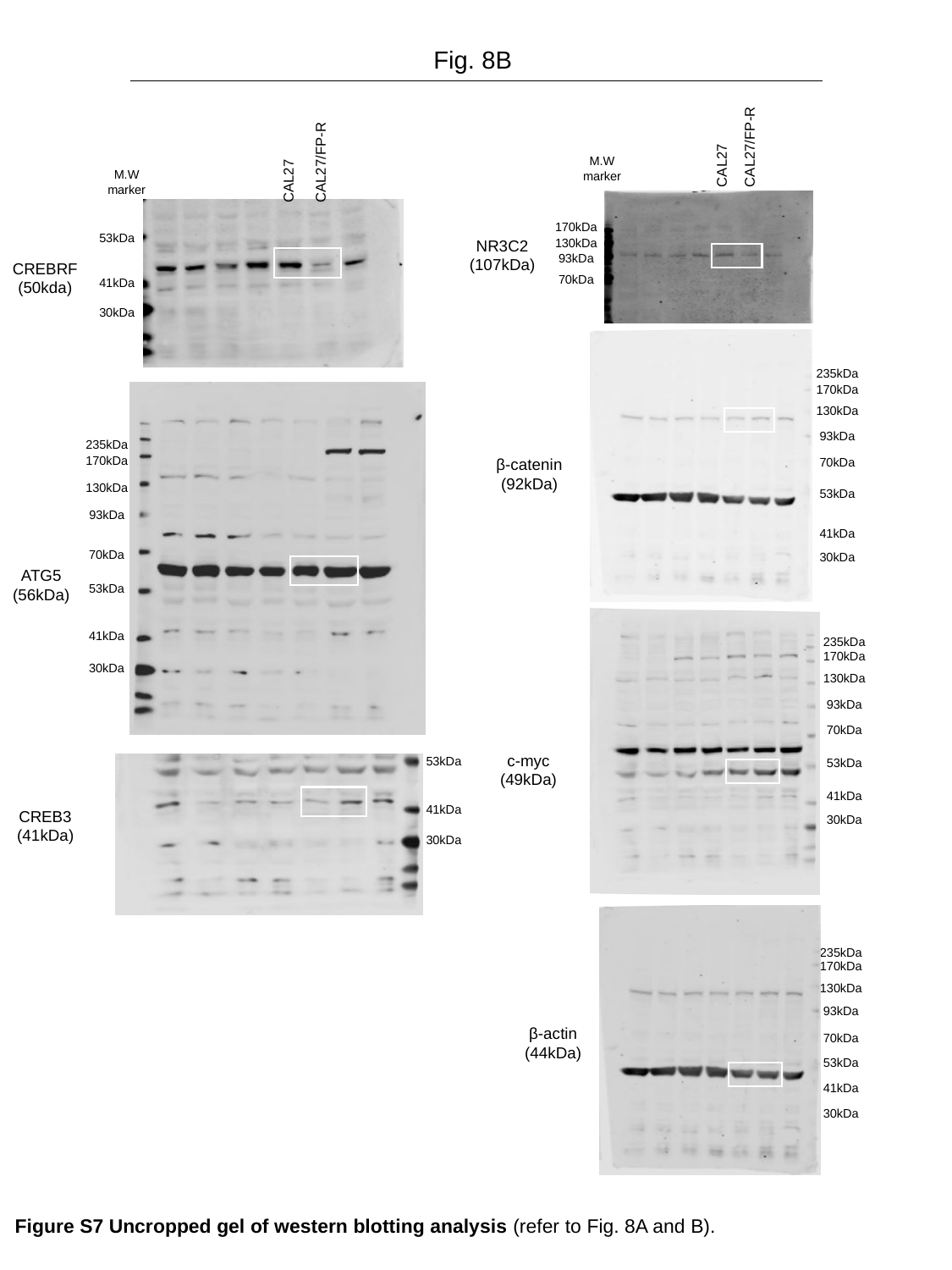

Fig. 8B
CAL27/FP-R
CAL27
CAL27/FP-R
CAL27
M.W marker
M.W marker
170kDa
53kDa
130kDa
NR3C2
(107kDa)
93kDa
CREBRF
(50kda)
70kDa
41kDa
30kDa
235kDa
170kDa
130kDa
93kDa
235kDa
170kDa
β-catenin
(92kDa)
70kDa
130kDa
53kDa
93kDa
41kDa
70kDa
30kDa
ATG5
(56kDa)
53kDa
41kDa
235kDa
170kDa
30kDa
130kDa
93kDa
70kDa
c-myc
(49kDa)
53kDa
53kDa
41kDa
41kDa
CREB3
(41kDa)
30kDa
30kDa
235kDa
170kDa
130kDa
93kDa
β-actin
(44kDa)
70kDa
53kDa
41kDa
30kDa
Figure S7 Uncropped gel of western blotting analysis (refer to Fig. 8A and B).

## Slide 12
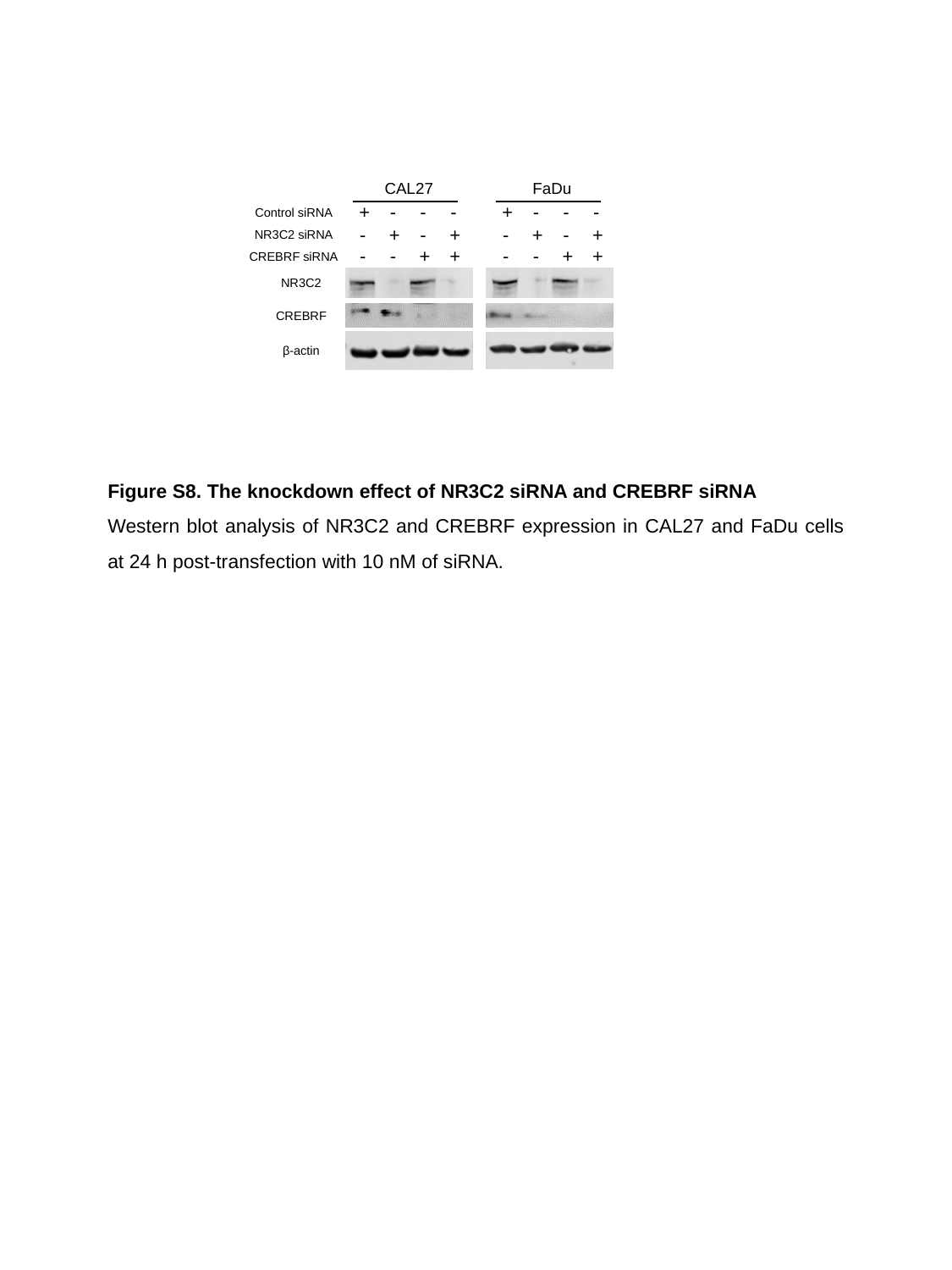

CAL27
FaDu
+
-
-
-
+
-
-
-
Control siRNA
-
+
-
+
-
+
-
+
NR3C2 siRNA
-
-
+
+
-
-
+
+
CREBRF siRNA
NR3C2
CREBRF
β-actin
Figure S8. The knockdown effect of NR3C2 siRNA and CREBRF siRNA
Western blot analysis of NR3C2 and CREBRF expression in CAL27 and FaDu cells at 24 h post-transfection with 10 nM of siRNA.
